# Two Primary Standards for Low Flows of Gases

**DOI:** 10.6028/jres.109.031

**Published:** 2004-08-01

**Authors:** Robert F. Berg, Stuart A. Tison

**Affiliations:** National Institute of Standards and Technology, Gaithersburg, MD 20899-8364; Mykrolis Corporation, Allen, TX 75013-8003

**Keywords:** constant pressure, gas flow meter, gravimetric, laminar flow meter, nitrogen, primary standard, volumetric

## Abstract

We describe two primary standards for gas flow in the range from 0.1 to 1000 μmol/s. (1 μmol/s ≅ 1.3 cm^3^/min at 0 °C and 1 atmosphere.) The first standard is a volumetric technique in which measurements of pressure, volume, temperature, and time are recorded while gas flows in or out of a stainless steel bellows at constant pressure. The second standard is a gravimetric technique. A small aluminum pressure cylinder supplies gas to a laminar flow meter, and the integrated throughput of the laminar flow meter is compared to the weight decrease of the cylinder. The two standards, which have standard uncertainties of 0.019 %, agree to within combined uncertainties with each other and with a third primary standard at NIST based on pressure measurements at constant volume.

## 1. Introduction

Integrated circuits are made in reaction chambers, or process “tools”, each of which receives gases from several mass flow controllers at rates from 1 to 10 000 μmol/s. (1 μmol/s ≅ 1.3 cm^3^/min at 0 °C and 1 atmosphere.) Flow uncertainties are typically 1 % at present, but improvements to 0.5 % are desired [1,2]. The role of the National Institute of Standards and Technology in achieving such improvements is to provide accurate primary standards for gas flow for the semiconductor industry, especially manufacturers of mass flow controllers. This paper describes two such standards whose uncertainty achieves the industry goal of 0.025 % [1,2].

The first primary standard, which is based on measurements of pressure, volume, temperature, and time, is a constant-pressure flow meter (CPFM). Operation at constant pressure eliminates problems due to adiabatic heating or cooling that can appear in a constant-volume (pressure-rate-of-rise) technique. The CPFM is similar to a vacuum standard used at NIST [3] in that it inserts a piston into an oil-filled chamber; however, the piston is much larger and its drive train can handle pressures up to 900 kPa.

The second primary standard, the gravimetric flow meter (GFM), is an adaptation of techniques used in industry to calibrate commercial laminar flow meters [4] and in the NIST Gas Metrology Group to create accurately known gas mixtures. The GFM uses an electronic mass comparator to weigh a gas cylinder before and after a gas flow. The change of weight effectively calibrates a laminar flow meter (LFM) [5] whose measurements can be integrated to high accuracy. This technique is “static”; a “dynamic” gravimetric technique measures the cylinder’s mass while the gas is flowing.

Both flow standards have standard uncertainties of 0.019 %. (All uncertainties are reported as standard relative uncertainties corresponding to a coverage factor *k* = 1). We verified their trustworthiness by comparing them to each other and to a third primary flow standard based on pressure measurements at constant volume [6]. Sections 2 and 3 describe the construction, operation, and uncertainty of the CPFM and GFM respectively. Section 4 describes the comparisons of the CPFM and GFM with each other and with the third primary flow standard.

## 2. Constant-Pressure Flow Meter (CPFM)

### 2.1 Principle of Operation

**Fig. 1:**
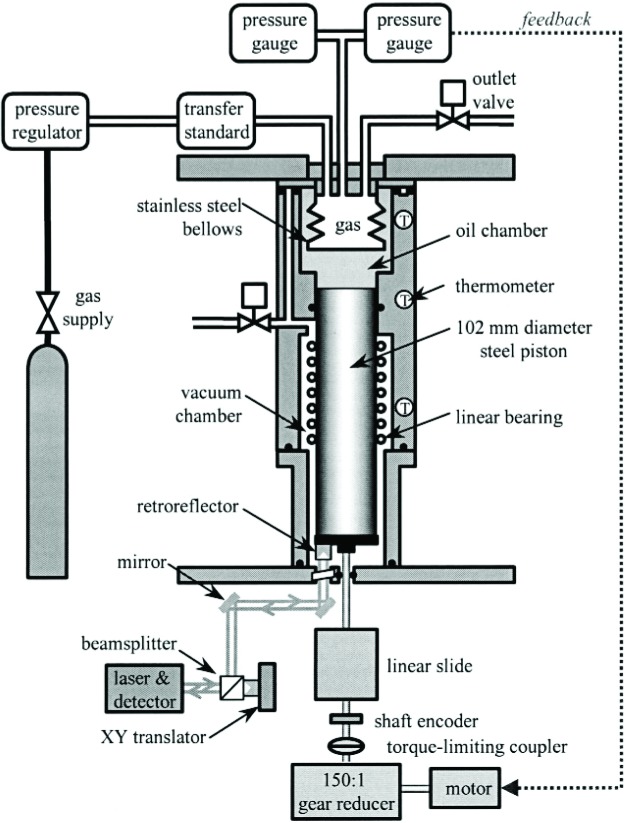
Schematic diagram of the CPFM. Moving the transfer standard from the input to the outlet changes the CPFM from a flow sink to a flow source. The feedback pressure gauge needs to be sensitive but not accurate.

Figure 1 is a schematic diagram of the CPFM. Its largest moving part is a piston that moves into or out of an oil-filled chamber. Consequently, gas flows out of or into a metal bellows contained in the oil chamber. A displacement Δ*x* of the piston out of the oil chamber increases the bellows volume by (π *D*^2^/4) Δ*x*, where *D* is the piston diameter. If the gas pressure *P* in the bellows remains constant, the number of moles of gas in the bellows increases by Δ*n*, and the average flow rate during the interval Δ*t* is

$${\dot{n}}_{\text{CPFM}}=\frac{\Delta n}{\Delta t}=\frac{P}{R_{\text{gas}}T\left(1+B_{P}P\right)}\frac{\pi D^{2}\Delta x}{4\Delta t},$$ (1)

where *R*_gas_, *T*, and *B_P_* are the universal gas constant, the gas temperature, and the gas’s second pressure virial coefficient, respectively.

During the flow measurement depicted in Fig. 1 the CPFM acts as a flow sink, and gas flows at a constant rate through the flow meter to be calibrated (transfer standard) and into the CPFM. (Moving the transfer standard from the input to the exhaust changes the CPFM from a flow sink to a flow source.) Eq.(1) is used periodically calculate the amount of gas accumulated in the CPFM bellows, which is compared to the integrated molar flow rate through the transfer standard.

### 2.2 Mechanical Components

The piston (102 mm diameter and 406 mm length) was ground from A-6 tool steel with a root-mean-square surface finish of 0.2 μm. A coordinate measuring machine determined its diameter variations. The bellows is a commercially available, edge-welded, stainless-steel, vacuum bellows, with a spring constant of 1.2 kN/m. The maximum volume defined by its effective diameter (124 mm) and stroke (86 mm) is 1.04 L. Most of the bellows stroke can be used for flow measurements because the piston’s diameter and allowed stroke (110 mm) define a swept volume of 0.90 L. (Δ*n* = 0.036 mol at 100 kPa.)

The piston’s drive train begins with a 120 W DC motor whose speed is reduced by a 150:1 gear reducer. The reducer drives a linear slide through a torque-limiting flexible coupling. The coupling prevents the application of a large torque to the linear slide that would be caused by binding of the piston or the drive train. The linear slide is a large translation stage that converts rotation to vertical displacement. A carriage attached to the slide drives a 19 mm diameter shaft that ends in a radial bearing attached to the piston’s base. A ball bushing linear bearing constrains the piston’s vertical motion. By design, the drive train can handle gas pressures to nearly 1000 kPa (the associated force on the piston corresponds to the weight of a of 830 kg piston). In practice, the coupling, which was designed to limit torques to 11 N · m, slipped at pressures greater than 900 kPa.

The upper end of a thick-walled aluminum housing forms the oil chamber. The lower end forms a vacuum chamber that contains the piston and linear bearing. Rubber O-rings seal the housing against thick upper (37 mm) and lower (25 mm) aluminum support plates. A third plate (25 mm) supports the linear slide. All three plates are supported by a welded aluminum frame via leveling adjustments. The aluminum frame also supports the CPFM’s vacuum manifold, laser interferometer, and electronic components.

The oil chamber holds approximately 1 L of diffusion pump oil (“Octoil S” or di 2 ethylhexyl sebacate). Degassing of the oil is important. Gas bubbles in the oil add an incorrect time dependence to the apparent flow rate, especially at pressures below 100 kPa. This occurs because the volume of a gas bubble depends on the oil pressure, and the oil pressure differs from the pressure of the gas in the bellows due to the spring constant of the bellows. Filling the oil chamber involves draining degassed oil from a diffusion pump into the evacuated chamber. The evaporation and boiling-induced stirring that occur during normal operation of the diffusion pump assist the removal of dissolved air. The higher temperatures assist the removal of volatile contaminants from the oil.

The design of the oil chamber minimizes the likelihood of an air leak into the degassed oil. The oil chamber’s lower end is a rubber O-ring that provides a sliding seal for the piston to prevent oil from leaking into the vacuum chamber. A gas-tight seal is not required because the vacuum chamber acts as a “guard” vacuum. The oil chamber’s upper end is sealed from atmosphere by two concentric O-rings. In between the two O-rings at intermediate radius is a groove that is linked to the vacuum chamber. The groove therefore is a guard vacuum space for the upper seal. Evacuation of the vacuum chamber also eliminates atmospheric corrections to the interferometer’s measurements of piston displacement.

### 2.3 Measurements of Displacement, Pressure, Temperature, and Time

The piston’s displacement is measured by a commercial laser interferometer. A retroreflector attached to the beamsplitter defines the reference beam. A retroreflector attached to the bottom of the piston defines the displacement beam. The two beam-steering mirrors give the degrees of freedom needed to align the displacement beam over the full travel (110 mm) of the piston. The *XY* translator aligns both beams with the photodetector. The interferometer’s internal software assumes that the displaced retroreflector moves through air at standard conditions. Instead, it moves through vacuum, so a correction for the index of refraction of air is made later during the analysis.

Feedback control of the bellows pressure requires a sensitive gauge with an analog output voltage. Its uncertainty is unimportant if a separate, accurate gauge is used to record pressure. Most flows are measured near 100 kPa, so the feedback gauge is usually a metal diaphragm capacitance transducer whose full scale pressure is only 133 kPa. Similarly, the recording gauge is either a 133 kPa quartz Bourdon gauge or a 310 kPa quartz resonant gauge. Similar models designed for higher pressures are used for pressures up to 1000 kPa.

A multimeter reads the four-terminal resistances of five platinum resistance thermometers (PRTs) that are imbedded in the wall of the aluminum housing. Three of the PRTs are located at the uppermost level indicated in Fig. 1. Their temperatures are approximately equal to that of the gas in the nearby bellows.

A sealed box constructed from 5 mm thick foam board encloses the aluminum housing and the adjacent part of the pressure manifold. The air in the box is heated and stirred by a thermoelectric/fan unit whose power supply controls the temperature measured by a thermistor attached to the aluminum housing. This scheme holds the CPFM temperature near 24 °C, and it suppress variations driven by room temperature (typically 0.2 K) by a factor of 30.

Time is read from the computer’s system clock.

### 2.4 Gas Handling

The CPFM’s gas manifold includes the flow path and the vacuum plumbing. The flow path comprises the bellows, the pressure gauges, and various manual and pneumatic valves. Connections are made through stainless steel tubing with deformable metal gasket seals. The connection between the transfer standard and the CPFM is a capillary whose 1.3 mm inner diameter minimizes the connecting volume while presenting a tolerable flow impedance. The vacuum plumbing includes more valves, a vacuum gauge, and an air-cooled drag pump that is used to evacuate the bellows and to provide a pressure reference for the quartz Bourdon gauge. An oil-sealed mechanical pump backs the drag pump; a second mechanical pump evacuates the vacuum chamber.

The top space of the bellows and the plumbing between the bellows and the pressure gauges create a 0.2 L “dead” volume. This volume was measured by analyzing measurements of pressure as a function of piston displacement.

### 2.5 Electronic Control

Custom electronic circuitry controls the piston’s speed in either a manual or a pressure-feedback mode by sending appropriate signals to the motor’s controller. In the manual mode, the piston can be raised and lowered at speeds from 0.001 mm/s to 0.5 mm/s. In the feedback mode, the voltage output of the pressure gauge is compared to a reference voltage. Usually, the reference voltage is the latched value of the pressure gauge’s output at the beginning of the piston stroke. The difference between the gauge and reference voltages is amplified by an analog circuit whose gain and integration time constant can be varied from the control panel.

The control panel includes a simple bar graph display of the piston’s position, which is inferred from 10 optical sensors that read the position of the linear slide. Three devices protect the CPFM against over-extension of the piston in the following order. (1) Sensors at the lowest and highest positions stop the motor drive signal. (2) Mechanical switches turn off the motor current. (3) Mechanical stops prevent motion of the linear slide.

Custom software periodically records measurements of the time, piston displacement, pressure, PRT temperatures, and the flow rate reported by the transfer standard. The measurement interval varies from 6 s to 60 s, depending on piston speed. Communication with the laser interferometer via RS-232 required custom dynamic link libraries (DLLs) supplied by the interferometer company. An earlier version of one of the DLLs caused an interferometer error of 0.05 %, which caused the CPFM data to be offset from the GFM data by 0.05 %. However, the cause of the offset was found only when the DLL was upgraded for other reasons. Approximately half of the CPFM data shown in Fig. 9 required correction for this error.

### 2.6 Operation and Data Analysis

Operation of the CPFM requires procedures each day and for each run, defined as one stroke of the piston. The following procedures assume that the CPFM acts as a flow sink; acting as a flow source requires starting the piston at the bottom.

#### CPFM Daily Procedure

1. Zero the quartz Bourdon gauge.
2. Flush and pump to remove any contaminating gas from previous runs.
3. Evacuate the vacuum chamber.
4. Move the piston to its lowest position and zero the laser interferometer’s output.

#### CPFM Run Procedure

1. Move the piston to its highest position.
2. With the exhaust valve open, establish a steady flow through the transfer standard and the CPFM.
3. Sample and hold the output of the feedback pressure gauge.
4. Close the exhaust valve to divert the flow into the bellows.
5. Open the exhaust valve when the piston reaches the lowest position.

The data are stored in ASCII files and analyzed in a spreadsheet. The analysis includes the following for each time step.

#### CPFM Analysis Procedure

**Fig. 2:**
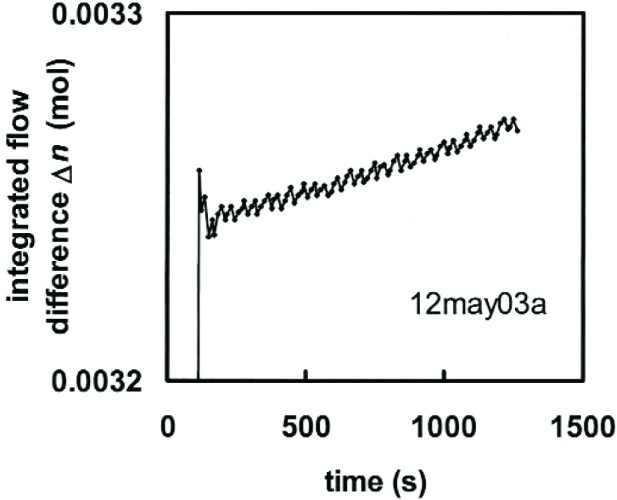
Difference between the integrated flow through the transfer standard and the number of moles in the CPFM during one run at 29 μmol/s. The flow reported by the transfer standard is greater than the flow into the CPFM by the slope of 0.023 μmol/s. The sawtooth amplitude corresponds to 10 μm of stick-slip motion of the piston.

1. Calculate the number of moles *n*_CPFM_(*t*) in the CPFM at time *t*.
2. Integrate the molar flow rate *ṅ*_transfer_ to obtain the number of moles *n*_transfer_(*t*) that flowed through the transfer standard since the beginning of the run.
3. Plot the molar difference Δ*n*(*t*) ≡ *n*_transfer_(*t*)− *n*_CPFM_(*t*). See Fig. 2.
4. Define the start and stop times *t*start and *t*_stop_ by the interval during which Δ*n*(*t*) is linear in time.
5. The apparent difference in flow rates is the slope of Δ*n* during the interval from *t*_start_ to *t*_stop_, namely

$${\dot{n}}_{\text{transfer}}-{\dot{n}}_{\text{CPFM}}=\frac{d\Delta n}{dt}.$$ (2)

### 2.7 Uncertainty

Most of the following uncertainties were calculated from Eq.(1). Unless stated otherwise, they assume that the CPFM is operated at the usual pressure of 100 kPa absolute.

#### Piston Cross Sectional Area

The flow rate is proportional to the piston’s cross sectional area, which was determined with a coordinate measuring machine at (20.0 ± 0.5) °C. Diameters were measured at various heights along the piston in two runs. The measurement uncertainty, the surface roughness, and the difference between the averages of the two runs were negligible compared to the difference, *δD*_max_ = 1.4 μm, between the maximum and minimum measurements within an individual run.

Thermal expansion and the 0.5 K temperature uncertainty during the diameter measurements contribute an uncertainty of *δD_T_* = 0.6 μm. The relative flow uncertainty due to the piston’s area is thus

$$u_{\text{area}}=2{\left[{\left(\frac{\delta D_{\max}}{D}\right)}^{2}+{\left(\frac{\delta D_{T}}{D}\right)}^{2}\right]}^{1/2}=0.003\%.$$ (3)

#### Piston Displacement

The interferometer manufacturer specified an accuracy of “1 part per million” for averaged measurements. However, the interferometer’s repeatability limits the accuracy of a displacement measurement of the moving piston. Measurements made of the motionless piston during a 300 s interval had a standard deviation of *δx* = 0.3 μm. The displacement contribution to the relative flow uncertainty is

$$u_{x}=\frac{\delta x}{\Delta x}=0.0003\%,$$ (4)

which is negligible.

We made a direct check of the volume displacement by filling the bellows and the adjacent manifold with water and connecting the manifold to a flask on a mass balance. The piston was then stroked up and down three times; each stroke added or removed water from the flask. The average relative difference between the volume *V*_mass_ corresponding to the mass change and the volume *V*_calc_ calculated from the piston area and the interferometer displacement was *V*_mass_/*V*_calc_ − 1 = − (0.014 ± 0.009) %. (The difference from zero is significant. A possible cause was a 50 cm^3^ air bubble trapped in the upper end of the bellows. The change of hydrostatic pressure during a piston stroke would have changed the bubble volume by 0.10 cm^3^, or 0.14 % of the displacement.)

#### Time

The flow rate is inversely proportional to the time interval Δ*t* between the first and last piston displacements used in the analysis. The accuracy of the computer’s clock was estimated by comparing it to a national time standard (www.time.gov) many times during a one-month interval. The relative errors in the elapsed time *δt*_clock_ were always less than 0.004 %.

During each measurement cycle, the time assigned to the piston’s displacement measurement is the clock reading that occurs immediately before. The interval Δ*t*_reading_ between the measurement and the clock reading is unimportant, but random variation of Δ*t*_reading_ adds to the flow uncertainty. The difference between clock readings immediately before and after the displacement measurement was found to be less than the clock resolution of 0.01 s, so the variation is *δt*_reading_ < 0.01 s.

The largest flow possible at one atmosphere is limited by the piston’s maximum speed. At 100 μmol/s the associated piston travel time is only Δ*t* ≅ 300 s, so the relative flow uncertainty due to *δt*_reading_ is 0.003 %. Operating at larger pressure increases Δ*t* and reduces this uncertainty. In general, the relative flow uncertainty due to time is

$$\begin{array}{l} u_{\text{time}}={\left[{\left(\frac{\delta t_{\text{clock}}}{\Delta t}\right)}^{2}+{\left(\frac{\delta t_{\text{reading}}}{\Delta t}\right)}^{2}\right]}^{1/2}\\ ={\left[{\left(0.004\%\right)}^{2}+{\left(\frac{100\text{kPa}}{P}\right)}^{2}{\left(\frac{\dot{n}}{100\mu \text{mol/s}}\right)}^{2}{\left(0.003\%\right)}^{2}\right]}^{1/2}. \end{array}$$ (5)

#### Temperature

The PRTs were calibrated with an uncertainty of approximately *δT*_cal_ = 0.01 K, which can be small in comparison with the difference between the temperature of the gas in the bellows and the temperature of the aluminum housing that holds the PRTs. One cause of that difference is the heating or cooling that follows a large pressure change, after which the gas temperature decays to that of the surrounding oil. The decay time constant, which was inferred from observations of the pressure’s time dependence with zero flow, is approximately 12 min. A corresponding delay between a pressure change and the beginning of a flow measurement makes the pressure-induced temperature difference negligible.

Another cause of the temperature difference between the gas and the PRTs appears to be a time lag between the oil temperature and the room temperature. This lag, which has been observed only at very small flow rates, was estimated by making flow and temperature measurements during a 3 day interval during which gas flowed at only 0.2 μmol/s through the LFM into the CPFM. The difference between the integrated CPFM and LFM flow rates had a time dependence that was similar to that of the PRT temperature, except that it lagged the temperature by approximately 1 h. For a typical drift of 0.01 K/h, the time lag of one hour causes a temperature difference between the gas and the PRTs of *δ T*_gas_ = 0.01 K.

The differences among the PRTs due to calibration drift or to temperature gradients on the wall of the oil chamber are *δ T*_wall_ = 0.03 K or smaller. Differences of temperature between the connecting tubing and the aluminum housing are negligible due to the tubing’s relatively small volume. The relative flow uncertainty assigned to temperature is thus

$$u_{T}={\left[{\left(\frac{\delta T_{\text{gas}}}{T}\right)}^{2}+{\left(\frac{\delta T_{\text{wall}}}{T}\right)}^{2}\right]}^{1/2}=0.011\%.$$ (6)

#### Pressure

The 310 kPa quartz flexure gauge was specified by the manufacturer to be accurate for one year to 31 Pa (0.01 % full scale). The gauge was recalibrated by comparing it to a piston gauge, so that its accuracy shortly afterwards was limited by hysteresis and by the deviation of the gauge’s output from its description by a cubic polynomial. Both quantities were about 5 Pa; their quadrature sum yielded the gauge’s calibration accuracy *δ P*_calibration_ = 7 Pa. The 133 kPa quartz Bourdon gauge was then calibrated by comparing it to the recently calibrated flexure gauge. Quartz Bourdon gauges have calibration drifts that are typically smaller than 0.01 % full scale per year, or *δ P*_drift_ = 13 Pa. At atmospheric pressure, the contribution of pressure to the relative flow uncertainty is thus

$$u_{P}=\left[{\left(\frac{\delta P_{\text{calibration}}}{P}\right)}^{2}+{\left(\frac{\delta P_{\text{drift}}}{P}\right)}^{2}\right]=0.015\%.$$ (7)

For P > 133 kPa, a gauge with a full-scale pressure of 2.8 MPa was used. This increased the relative uncertainty to approximately *u*_P_ ≈ 280/*P*. (A more optimum gauge with a full-scale pressure of only 1 MPa would have contributed only *u*_P_ ≈ 100/*P*.)

#### Oil Expansion

An increase of room temperature decreases the oil density. This effect increases the apparent flow rate by

$$\begin{array}{c} {\dot{n}}_{\text{oil}}=\left(\alpha_{\text{oil}}V_{\text{oil}}-\alpha_{\text{chamber}}V_{\text{chamber}}\right)\frac{P}{R_{\text{gas}}T}\frac{dT}{dt}\\ ≅\left(31\frac{\mu \text{mol}}{K}\right)\left(\frac{P}{100\text{kPa}}\right)\frac{dT}{dt}, \end{array}$$ (8)

where *V*_oil_ and *V*_chamber_ are the respective volumes of the oil and the aluminum oil chamber and *α*_oil_ [7] and *α*_chamber_ are the respective volume expansivities. A typical temperature drift of d*T*/d*t* = 0.01 K/h causes an uncertainty of

$$u_{\text{oil}}=\frac{{\dot{n}}_{\text{oil}}}{\dot{n}}=\frac{8\times 10^{-5}\mu \text{mol/s}}{\dot{n}},$$ (9)

which is negligible for flows greater than 1 μmol/s.

The effect of oil expansion on flows less than 1 μmol/s also is negligible if the measurement uses the full piston stroke. Such measurements require at least Δ*t* = Δ*n/*ṅ = 10 h, during which d*T*/d*t* typically changes sign. Limiting Δ*t* to much less than 10 h by using only part of the piston stroke greatly increases the contribution of *u*_oil_.

#### Other Sources of Uncertainty

For the nitrogen measurements reported here, the uncertainty of the gas’s equation of state is negligible. An exception may occur for gases such as SF_6_, whose second pressure virial coefficient *B*_P_ is 55 times larger than that of nitrogen. A recent careful study of the properties of SF_6_ [8] does not state the uncertainty of *B*_P_ directly; however inspection of deviation plots in [8] suggests that it is roughly 1 % near 300 K. The resulting contribution to the flow uncertainty would be as large as 0.01 % at atmospheric pressure. Operation with gases such as SF_6_ at higher pressures would require use of the third virial coefficient in the model of Eq.(1).

The effect of an impurity is negligible because the molar volume depends only weakly on composition. For example, in the unlikely event that the nitrogen had an unknown impurity of 1 % SF_6_, the resulting error would be only 0.01 % at atmospheric pressure.

A drift of room temperature will cause the tubing between the transfer standard and the CPFM to be an apparent source or sink of flow. The small volume of the connecting capillary makes this effect negligible.

#### Total Uncertainty

The total relative flow uncertainty is

$$u_{\text{CPFM}}={\left(u_{\text{area}}^{2}+u_{\text{time}}^{2}+u_{x}^{2}+u_{T}^{2}+u_{P}^{2}+u_{\text{oil}}^{2}\right)}^{1/2}.$$ (10)

**Fig. 3:**
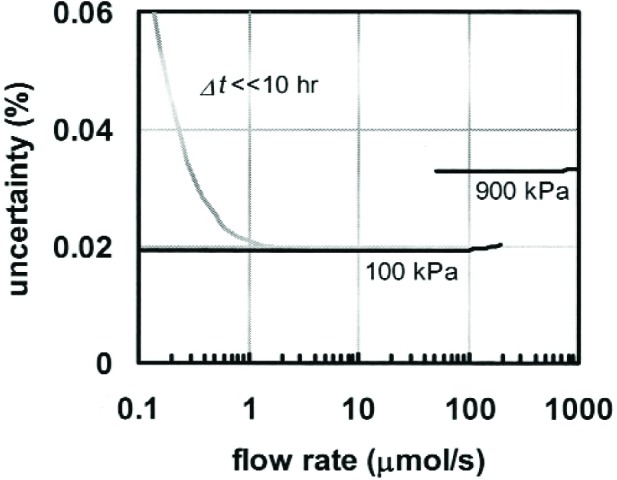
Relative uncertainty of the CPFM as a function of flow rate. Small flow rates have small uncertainty if the measurement time is at least 10 hours. Operation at the maximum practical pressure of 900 kPa enabled flow rates up to 1000 μmol/s but was implemented with a 2.8 MPa pressure gauge with greater uncertainty. (Use of a 1 MPa gauge would have decreased the 900 kPa curve to 0.02 %.)

Figure 3 plots the relative uncertainty as a function of flow rate. At the smaller flow rates, the uncertainty is dominated by *u*_oil_ due to thermal expansion of the oil only if the measurement time is limited. Otherwise, *u*_oil_ is negligible. At the larger flow rates, the uncertainty is dominated by *u*_P_ due to the gauge used to measure the necessarily greater bellows pressure. For flow rates less than 100 μmol/s, the standard uncertainty is *u*_CPFM_ = 0.019 %.

## 3. Gravimetric Flow Meter (GFM)

### 3.1 Principle of Operation

**Fig. 4:**
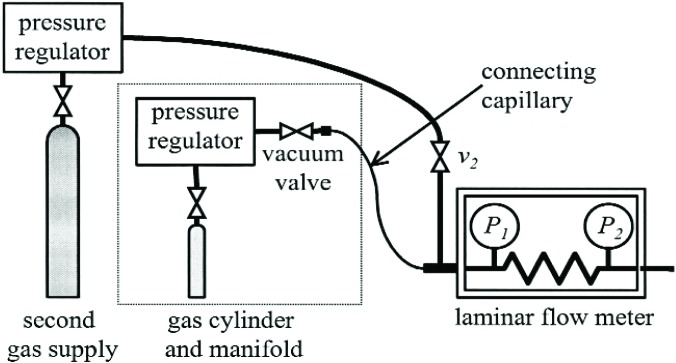
Schematic of the gravimetric flow meter.

Figure 4 is a schematic of the GFM. During a gravimetric flow measurement, gas flows from a small gas pressure cylinder through a laminar flow meter. The mass change of the gas cylinder is compared to the integral of the mass flow rate through the laminar flow meter. The weight *w*_ref_ of a reference cylinder is measured as well as the weight *w*_gas_ of the gas cylinder; this allows calculation of the change of mass from the change of the difference *w*_gas_ − *w*_ref_. The reference cylinder’s similar mass reduces errors due to drift of the balance, and its similar volume eliminates the need for buoyancy corrections.

An integrating flow meter is required to compare a flow rate to a mass change. The laminar flow meter used here for that purpose is used also to calibrate other flow meters [5]. It is therefore a transfer standard as well as an essential part of the GFM.

### 3.2 Components

#### Gas Cylinder

**Fig. 5:**
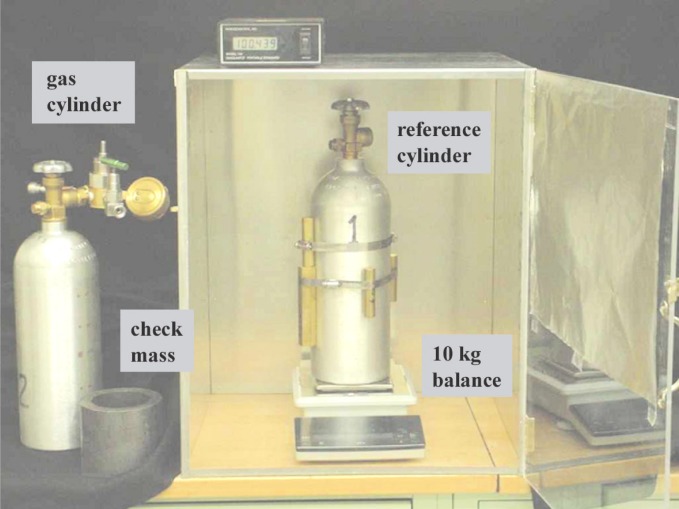
Weighing apparatus used for the GFM.

Both aluminum cylinders (Luxfer)^1^ (see Fig. 5) are rated for a maximum working pressure of 12.4 MPa (1800 psi). Their height of 460 mm includes a conventional brass valve and outlet, and their empty mass is approximately 3.5 kg. The outside surfaces of the cylinders were cleaned with water and detergent after removing all labels. Plastic parts such as valve handles were removed from both cylinders and from the pressure regulator. Some plastics adsorb water when the room humidity increases; the resulting weight variations could exceed 100 mg. (The small amounts of PTFE tape that were used to seal the NPT threads had a negligible effect. A sample of the tape was tested by weighing it before and after soaking it in water.)

The gas cylinder has a gas manifold that allowed the cylinder to be filled to high pressure and emptied to low pressure. The manifold comprised a metal-sealed pressure regulator (Tescom 44-5013-241)^1^ with the components listed in Table 1 connected to its female NPT fittings. With the manifold, the gas cylinder’s total weight is 5.2 kg.

The absence of leaks from the gas cylinder’s valve packing and the connection to the cylinder was verified by filling the tank to its working pressure, applying a detergent solution, and using a magnifying loupe to look for bubbles. The absence of an observable bubble implied that the leak rate was less than 2 × 10^−4^ μmol/s. The absence of leaks from the gas manifold was verified by connecting a mass-spectrometer leak detector to the VCR connection and spraying the outside with helium. The leak rate was less than 4 × 10^−8^ μmol/s. (See also Fig. 8.)

#### Reference Cylinder

The reference cylinder is similar to the gas cylinder, but it lacks the gas manifold, and brass weights were strapped onto the cylinder by steel hose clamps. The weights were chosen so that the two cylinders never differed by more than 250 g during the flow measurements. Matching the cylinder weights minimizes errors caused by the balance’s short-term drift.

Matching the volumes of the reference and gas cylinders to within 100 cm^3^ eliminates the need for a buoyancy correction because a 1 kPa drift of atmospheric pressure changes the mass difference by less than the 2 mg repeatability of the balance. Changes of room temperature are similarly negligible. A matching volume was easily achieved because the aluminum cylinder bodies had the same shape, and the brass weights attached to the reference cylinder had a density similar to that of the manifold components on the gas cylinder.

#### Electronic Balance

The electronic balance has a capacity of 10 kg, a resolution of 1 mg, and a repeatability of 2 mg. It uses an internal weight for self-calibration. A windshield box is necessary (see Fig. 5). Most of the inside of the windshield’s clear plastic door was covered by aluminum foil to eliminate forces due to static electricity.

The gauge on the gas cylinder’s gas manifold protrudes 190 mm horizontally from the center line of the cylinder. This asymmetry gave the balance reading an orientation dependence (“corner load error”). Rotating the cylinder between weighings caused the apparent weight to vary smoothly with orientation; the difference between minimum and maximum was 30 mg. All weighings were done at the orientation that yielded the minimum apparent weight. Using the same orientation eliminates the orientation dependence from the mass differences. Using a slightly different orientation has a minimum effect because the orientation is near an extremum. Variations of the orientation were estimated to contribute less than 1 mg to variations of the apparent weight.

#### Laminar Flow Meter

The LFM, which is described in detail elsewhere [5], measures the temperature, entrance pressure, and exit pressure of gas flowing through a quartz capillary flow element. A hydrodynamic model converts these measurements into a molar flow rate. The model’s accuracy is limited by the uncertainty of its only free parameter, the capillary radius, which is determined by comparison against a primary flow standard. Calibration with the GFM means that the capillary radius is adjusted so that the integrated mass flow rate equals the mass change of the gas cylinder. In practice, one value of the capillary radius was used for all of the flows of each LFM flow element. The accuracy and stability of the LFM have been verified with nitrogen flow rates ranging from 0.1 to 1000 μmol/s [5]. The accuracy of its model was verified with four gases in addition to nitrogen.

### 3.3 Operation and Data Analysis

#### Filling the Gas Cylinder

Before filling the cylinder with a new gas, it is evacuated through the manifold’s vacuum valve. After the evacuation and subsequent filling, the cylinder valve remains open, so the gas flow is regulated only by the gas cylinder’s regulator and vacuum valve. Reducing the use of the cylinder valve reduces the likelihood of a leak past the valve packing.

The gas cylinder is filled by connecting its gas manifold to a supply cylinder with a high-pressure regulator. The high-pressure connecting line terminates with a male quick-connect fitting that matches the female fitting on the manifold. The high-pressure regulator is increased to approximately 11 MPa over an interval of 10 min; the slow filling keeps the gas cylinder’s temperature under 40 °C for safety. A typical filling is about 400 g, or 14 mol, of nitrogen.

An unknown impurity will cause errors with both parts of the gravimetric technique. The error in the LFM’s measurement is small because the effect of an impurity on gas viscosity is mild. For most gases, the LFM error will be less than 0.01 % if the impurity level is below 0.02 %, which is easily achieved. In contrast, the mass error can be larger because an unknown impurity with mole fraction *x_i_* and molecular weight *M_i_* will cause a relative flow error of approximately

$$\frac{\Delta \dot{n}}{\dot{n}}≅\frac{M_{i}-M}{M}x_{i}.$$ (11)

Two examples illustrate the care required to achieve a flow uncertainty of 0.01 % when *M* and *M_i_* differ greatly. Contamination of a helium flow by air must be approximately 0.002 % or less, and contamination of a nitrogen flow by SF_6_ (unusually heavy) must be approximately 0.002 % or less.

#### Flow Measurement

Each flow measurement uses the weight difference *w*_gas_ - *w*_ref_ before and after the gas flow. Obtaining the best accuracy requires following the procedure below as well as the recommendations of the balance manufacturer.

#### Weighing Procedure

1. Calibrate the balance daily.
2. Zero the balance.
3. Ensure that both cylinders are within 1 K of room temperature. This prevents air convection currents that change the cylinder’s apparent weight.
4. Place the reference cylinder on the balance and wait until the balance’s drift has stopped. (The present balance drifts typically 10 mg during the first 7 min after loading.)
5. Record *w*_ref_ at time *t*_1_. Exchange the reference and gas cylinders.
6. Record *w*_gas_ at time *t*_2_. Exchange the reference and gas cylinders.
7. Repeat steps 5 and 6 at least two more times. Do successive weighings at regular intervals (e.g., (*t*_2_ - *t*_1_) = (*t*_3_ - *t*_2_) = … = 60 s). The recovery of the balance from a large weight change is similar from weighing to weighing, so cyclic weighing approximately cancels the recovery out of the difference *w*_gas_ - *w*_ref_.
8. Average the three values for *w*_gas_ - *w*_ref_.

Measurements of other quantities are useful if the accuracy of the weighing technique is in doubt. These include the weight of a brass reference mass and measurements of ambient temperature, pressure, and humidity. Adding a small known mass to the cylinder will check the balance’s linearity.

The flow measurement itself requires the gas cylinder, the LFM or another flow meter whose output can be integrated accurately, a connecting capillary, and a second gas supply. See Fig. 4. The connecting capillary used for the present measurements is stainless steel with a VCR fitting at each end. Its inner diameter of 1.3 mm and length of 1 m presents a relatively small impedance to the flow while minimizing the volume between the gas cylinder’s manifold and the LFM. A small intermediate volume reduces the time required for the capillary’s pressure to decay to atmospheric after flow from the gas cylinder is stopped. The second gas supply is for flushing the connecting capillary.

#### GFM Run Procedure

1. Obtain the starting weight difference (*w*_gas_- *w*_ref_)start.
2. Attach the connecting capillary from the LFM to the gas cylinder’s vacuum valve. Use the second gas supply to pressurize the capillary while making the connection. This flushes the capillary and the small volume at the outlet of the vacuum valve.
3. Shut off the second gas supply (valve *v*_2_) and allow the pressure in the capillary and the flow meter to decay to atmospheric.
4. Open the bellow-sealed vacuum valve and adjust the regulator to obtain the desired flow rate.
5. After sufficient gas has flowed, close the bellows-sealed valve and allow the pressure in the capillary and the flow meter to decay to atmospheric.
6. Disconnect the capillary from the bellows-sealed valve.
7. Obtain the ending weight difference (*w*_gas_ - *w*_ref_)_stop_.

**Fig. 6:**
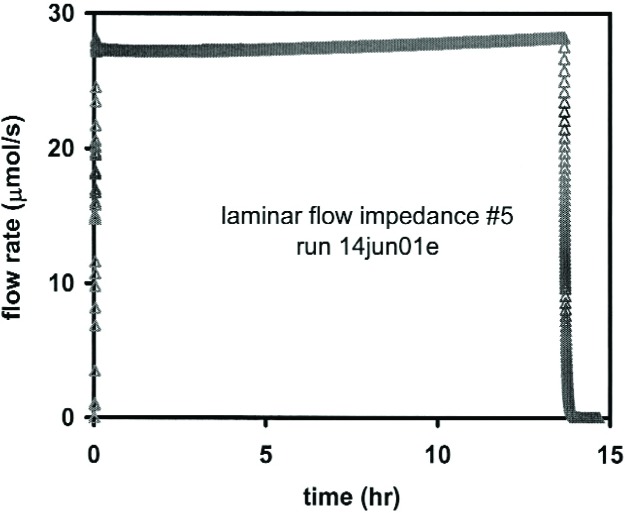
Flow through the laminar flow meter during a gravimetric flow measurement. Instability of the pressure regulator caused slow changes of the flow rate.

Figure 6 shows the flow rate through the LFM during a gravimetric flow measurement. Note the rapid changes in flow rate at the beginning and end of the run. Data were taken more frequently during these intervals by making faster, less accurate pressure measurements.

**Fig. 7:**
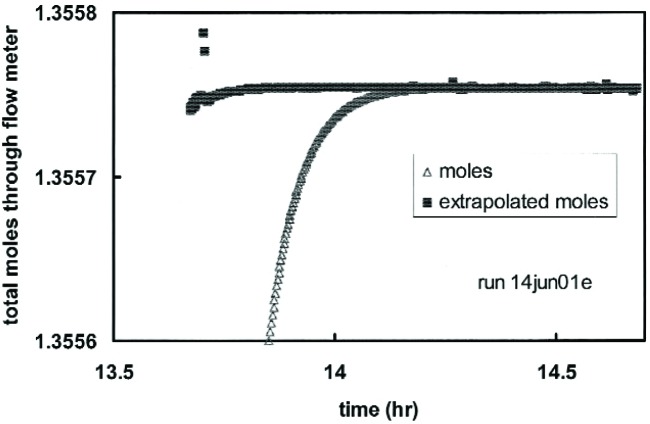
Extrapolation of the integrated flow through the laminar flow meter. The extrapolation yielded an estimate (“extrapolated moles”) that stabilized 0.4 h earlier than the integral obtained without extrapolation (“moles”).

Note also the decay of the flow rate at the end of the run caused by the return to atmospheric of the pressure in the connecting volume. For the flow element in Fig. 6, the final decay had a time constant of 4 min. However, the flow element for the smallest flows had a time constant of 40 min, so, after shutting the valve, hours were required for the flow rate to approach zero. We avoided such a long wait by assuming that the pressure in the connecting volume decayed exponentially to atmospheric. This allowed us to estimate the final integral of flow rate by extrapolation as follows. At time *t*, the total moles through the LFM, *n*_LFM_(*t*), is exponentially approaching its final value *n*_LFM_(∞). By assuming an exponential decay of pressure, the value of *n*_LFM_(∞) estimated at time *t* is

$$n_{\text{LFM}}\left(\infty \right)=n_{\text{LFM}}\left(t\right)-\left(\frac{P_{1}-P_{2}}{dP_{1}/dt}\right){\dot{n}}_{\text{LFM}}\left(t\right),$$ (12)

where *P*_1_ and *P*_2_ are the LFM’s input and output pressures, respectively. (*P*_1_ is also the pressure in the connecting volume.) Figure 7 illustrates the extrapolation by plotting *n*_LFM_(*t*) and *n*_LFM_(∞) for the run shown in Fig. 6. The total moles *n*_LFM_(*t*) reached its final value at time *t* = 14.2 h. The extrapolated moles *n*_LFM_(∞) stabilized at the same value but 0.4 h earlier. For the flow element for the smallest flows, the time savings was approximately 4 h.

#### Analysis

The number of moles removed from the gas cylinder is

$$n_{\text{GFM}}=\frac{{\left(w_{\text{gas}}-w_{\text{ref}}\right)}_{\text{start}}-{\left(w_{\text{gas}}-w_{\text{ref}}\right)}_{\text{stop}}}{M\left(1+\rho_{\text{air}}/\rho_{\text{brass}}\right)},$$ (13)

where *M* is the molecular weight, and *ρ*_brass_ and *ρ*_air_ are the densities of brass and air. (The weights *w*_gas_ and *w*_ref_ are described here in mass units.) The factor (1 + *ρ*_air_/*ρ*_brass_) = 1.000 15 accounts for the buoyancy correction that is implicit in the balance’s calibration. The balance yields the correct mass for a set of brass calibration weights because the buoyancy of each weight is proportional to its mass. In contrast, the buoyancy of the gas cylinder remains constant when its mass decreases.

The integrated flow through the LFM,

$$n_{\text{LFM}}=\int_{0}^{\infty }{\dot{n}}_{\text{LFM}}\left(t\right)dt,$$ (14)

is calculated by using the trapezoidal rule to sum the measurements of flow rate at discrete times during a finite interval, and the extrapolation described above is used for runs with long decay times.

The relative difference of the apparent flow rates is

$$\frac{{\dot{n}}_{\text{LFM}}-{\dot{n}}_{\text{GFM}}}{{\dot{n}}_{\text{GFM}}}\equiv \frac{n_{\text{LFM}}}{n_{\text{GFM}}}-1,$$ (15)

and the average flow rate is defined by

$$\langle {\dot{n}}_{\text{laminar}}\rangle \equiv \frac{\int_{0}^{n_{\text{laminare}}}{\dot{n}}_{\text{laminar}}\left(t\right)dn}{n_{\text{laminar}}}=\frac{\int_{0}^{\infty }{\dot{n}}_{\text{laminar}}^{2}\left(t\right)dt}{n_{\text{laminar}}}.$$ (16)

This definition weights the time-dependent flow rate by quantity of gas and not by time.

The volume of the gas cylinder decreases during a flow measurement due to the decrease of cylinder pressure. The volume decrease was estimated by measuring the cylinder’s diameter and height before and after a filling; adding 408 g of nitrogen filled the cylinder to 10 MPa and caused a volume change of Δ*V*_cylinder_ = 21 cm^3^. For nitrogen, the ratio of the change in the mass of displaced air to the mass of gas removed from the cylinder is therefore

$$\frac{\Delta m_{\text{air}}}{m_{\text{GFM}}}=\frac{\rho_{\text{air}}}{M}\frac{dV_{\text{bottle}}}{dn_{\text{GFM}}}=0.009\%.$$ (17)

For nitrogen, the result of Eq.(15) must be decreased by 0.009 %. For helium, the correction would be 0.063 %.

### 3.4 Uncertainty

#### Mass Change

The performance of the electronic balance limits the accuracy of the mass change measurements, so it was verified in two ways. First, calibration weights of 10 g, 100 g, and 300 g were added to the weight of the gas cylinder to check the balance’s linearity. The results were accurate to within the balance’s repeatability of *δ m*_repeat_ = 2 mg.

**Fig. 8:**
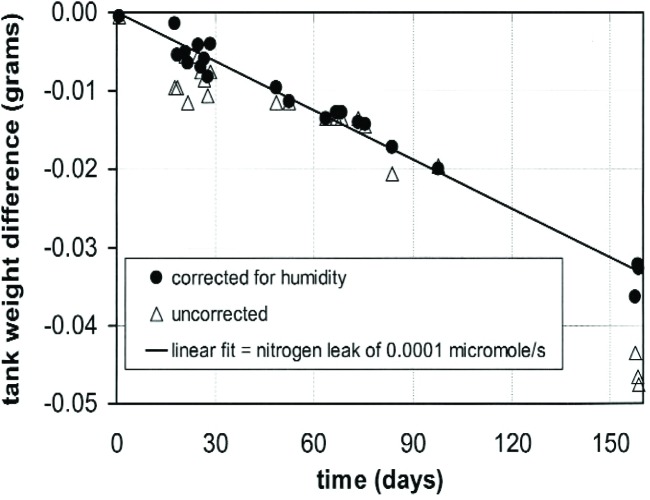
The weight difference of the gas and reference cylinders.

Second, the weight difference *w*_gas_ - *w*_ref_ was measured while the gas cylinder held 300 g of nitrogen. Figure 8 shows the results during a 5 month interval in which no flow measurements were made, but normal variations of humidity and pressure occurred. The results were fit to a linear function of humidity as well as time. The significant influence of relative humidity, which varied from 19 % to 62 %, was attributed to a difference in water adsorption between the surfaces of the gas and reference cylinders. In contrast, atmospheric pressure, which varied by 1.7 kPa, had no influence, as was expected from the matched volumes of the gas and reference cylinders.

The data were fit by a linear function of humidity and time. After correcting for the fitted value for humidity (0.35 mg per % relative humidity), the values of *w*_gas_ - *w*_ref_ in Fig. 8 can be described by a straight line whose slope corresponds to a nitrogen leak of 1 × 10^-4^ μmol/s. This apparent leak is 2 times smaller than the upper bound determined by the bubble check. The standard deviation of the differences between the corrected values and the fitted line is 1.5 mg, which is consistent with the balance’s repeatability.

The mass of gas that flows from the gas cylinder during a calibration is typically 40 g. The associated two weight determinations contribute a relative flow uncertainty of

$$u_{\text{mass}}=\frac{\sqrt{2}\delta m_{\text{repeat}}}{Mn_{\text{GFM}}}=\frac{\sqrt{2}\left(0.002g\right)}{\left(40g\right)}=0.007\%.$$ (18)

#### Humidity

The gravimetric flow measurements reported below did not use a humidity correction because the changes of relative humidity between weighings were 10 % or smaller. The estimated contribution to the relative flow uncertainty was therefore

$$u_{\text{humidity}}\leq \frac{\left(0.35\;\text{mg/\%}\right)\left(10\%\right)}{\left(40\;g\right)}=0.009\%.$$ (19)

#### Laminar Flow Meter

Variations of the lab temperature and the uncertainty and resolution of the LFM pressure measurements dominate the reproducibility of the LFM because those quantities can vary between flow measurements. Their quadrature sum is approximately 0.011 % [5].

The rapid changes of flow rate at the beginning and end of a GFM measurement also contribute to the reproducibility. Their contribution was estimated by flowing nitrogen through the LFM into the CPFM. In these tests, the moles accumulated in the CPFM (typically 0.3 mol) differed from the integrated LFM reading by 130 μmol or less. The corresponding uncertainty contributed to a typical GFM measurement was therefore less than (130 μmol)/(1.4 mol) = 0.009 %. Combining the two contributions to the LFM reproducibility yields

$$u_{\text{LFM reproducibility}}=0.014\%.$$ (20)

#### Clock Accuracy

The accuracy of the integral that yields *n*_LFM_ depends on the accuracy of the LFM’s timer. This is the same computer clock used for the CPFM, so the contribution of time to the relative flow uncertainty is

$$u_{\text{time}}=0.004\%.$$ (21)

#### Gas Purity

The manufacturer claimed that the nitrogen used in the present measurements had less than 0.001 % impurity. (This claim was consistent with a residual gas analysis up to 44 atomic mass units.) Assuming that likely impurities had molecular masses less than twice that of nitrogen gives an uncertainty contribution of

$$u_{\text{impurity}}\leq 0.002\%.$$ (22)

#### Total Uncertainty

For a typical gas mass of 40 g, the total uncertainty of the GFM,

$$\begin{array}{l} u_{\text{GFM}}={\left(u_{\text{mass}}^{2}+u_{\text{humidity}}^{2}+u_{\text{LFM reproducibility}}^{2}+u_{\text{time}}^{2}+u_{\text{im purity}}^{2}\right)}^{1/2}\\ \;=0.019\%, \end{array}$$ (23)

is limited chiefly by the reproducibility of the LFM.

## 4. Comparison of the Standards to Each Other and to a Third Standard

**Fig. 9:**
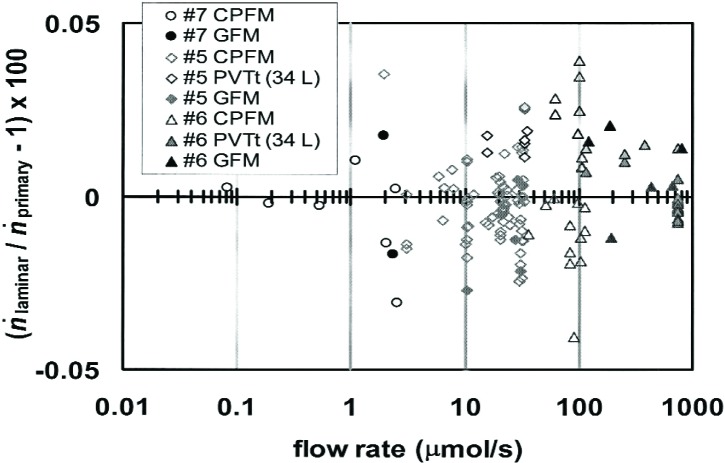
Figure 9. Comparison of the CPFM (open points) and the GFM (solid points) via the laminar flow transfer standard. Also shown is a similar comparison to a 34 L *PVTt* standard [6] based on pressure changes at constant volume.

The LFM was used as a transfer standard to compare the CPFM to the GFM. Laminar flow elements for small (#7), medium (#5), and large (#6) flows were used to span the range of flow from 0.08 to 800 μmol/s. Each element was assigned an effective radius that approximately minimized the deviations between the LFM and both primary flow standards. The use of a single radius for each element meant that the assignment did not affect the differences between the CPFM data and the GFM data. Each point on Fig. 9 represents (*ṅ*_LFM_/*ṅ*_primary_) −1, where “primary” denotes either CPFM or GFM. All of the CPFM flows were at 100 kPa, so the combined standard uncertainty (*k* = 1) for the comparison was

$$\begin{array}{l} u_{\text{CPFM \& GFM}}={\left(u_{\text{CPFM}}^{2}+u_{\text{GFM}}^{2}+u_{\text{LFM reproducibility}}^{2}\right)}^{1/2}\\ \;={\left[{\left(0.019\right)}^{2}+{\left(0.019\right)}^{2}+{\left(0.014\right)}^{2}\right]}^{1/2}\%\\ \;=0.030\%. \end{array}$$ (24)

The LFM (using the same effective radii) was used also to compare the CPFM to a larger *PVTt* flow standard at NIST [6]. This primary standard calculates flow rate from timing signals and the initial and final pressures in a 34 L, temperature-controlled tank. Overnight observation of the tank pressure revealed a small amount of outgassing. A corresponding correction of 0.0015 μmol/s was used to extend the lower range of the 34 L *PVTt* flow standard to 15 μmol/s. The combined standard uncertainty of the comparison was approximately

$$\begin{array}{l} u_{34\text{L PVTt \& CPFM}}={\left(u_{34\text{L PVTt}}^{2}+u_{\text{CPFM}}^{2}+u_{\text{LFM reproduci bility}}^{2}\right)}^{1/2}\\ \;={\left[{\left(0.014\right)}^{2}+{\left(0.019\right)}^{2}+{\left(0.014\right)}^{2}\right]}^{1/2}\%\\ \;=0.027\%. \end{array}$$ (25)

Figure 9 shows that the agreement among the three primary flow standards is consistent with the combined uncertainties of approximately 0.03 %. Figure 9 also indicates the stability of the flow standards; the data were taken during an interval of 2 years.

Table 2 gives the mean differences between the LFM and the primary flow standards. For all three flow ranges, the difference between any two flow standards is less than the combined uncertainty of the comparison. The standard deviations of the data are comparable to the combined standard deviations.

## 5. Conclusion

The two primary standards are in agreement despite their very different operating principles, thereby increasing confidence in their small uncertainty estimates. Because those uncertainties meet industry needs for at least the near future, improvements will be focused on improving the convenience of the standards.

## Figures and Tables

**Table 1:** Components of the gas cylinder’s manifold

Inlet #1	Nipple connection to cylinder with no check valve.
Inlet #2	High pressure shut-off valve & quick-connect fitting.
Outlet #1	Metal Bourdon pressure gauge.
Outlet #2	NPT/VCR adapter & bellows-sealed vacuum valve with VCR connections.

**Table 2:** Relative differences, in percent, between the LFM and the three primary flow standards. The first column gives the standard uncertainty of the primary flow standard. The other columns give the mean of the difference data shown in Fig. 9 and the standard deviation of the data. (The standard deviation of the mean value would be smaller by approximately the square root of the number of data.)

Primary flow standard	Small (#7) (0.08 to 2.55) μmol/s	Medium (#5) (2 to 33) μmol/s	Large (#6) (36 to 803) μmol/s
CPFM ± 0.019	−0.005 ± 0.014	−0.003 ± 0.010	+0.003 ± 0.020
GFM ± 0.019	+0.000 ± 0.024	−0.010 ± 0.013	+0.001 ± 0.020
large *PVTt* ± 0.014		+0.018 ± 0.006	+0.001 ± 0.006

## Footnotes

1 Certain commercial equipment, instruments, or materials are identified in this paper to foster understanding. Such identification does not imply recommendation or endorsement by the National Institute of Standards and Technology, nor does it imply that the materials or equipment identified are necessarily the best avialable for the purpose.
